# Different states of priority recruit different neural representations in visual working memory

**DOI:** 10.1371/journal.pbio.3000769

**Published:** 2020-06-29

**Authors:** Qing Yu, Chunyue Teng, Bradley R. Postle

**Affiliations:** 1 Department of Psychiatry, University of Wisconsin-Madison, Madison, Wisconsin, United States of America; 2 Department of Psychology, University of Wisconsin-Madison, Madison, Wisconsin, United States of America; Vanderbilt University, UNITED STATES

## Abstract

We used functional magnetic resonance imaging (fMRI) to investigate the neural codes for representing stimulus information held in different states of priority in working memory. Human participants (male and female) performed delayed recall for 2 oriented gratings that could appear in any of several locations. Priority status was manipulated by a retrocue, such that one became the prioritized memory item (PMI) and another the unprioritized memory item (UMI). Using inverted encoding models (IEMs), we found that, in early visual cortex, the orientation of the UMI was represented in a neural representation that was rotated relative to the PMI. In intraparietal sulcus (IPS), we observed the analogous effect for the representation of the location of the UMI. Taken together, these results provide evidence for a common remapping mechanism that may be responsible for representing stimulus identity and stimulus context with different levels of priority in working memory.

## Abstract 2

By tracking the neural representation of stimulus orientation and stimulus location while items were held at different levels of priority in visual working memory (“prioritized” and “unprioritized”), this study provides evidence that a common remapping operation may be engaged, selectively in the regions primarily responsible for representing feature-level information, when information held in working memory is outside the focus of attention.

## Introduction

Important for understanding the flexible control of behavior [1,2] is understanding working memory: the mental retention of task-relevant information and the ability to use it to guide contextually appropriate actions [3,4]. State-based theoretical models of working memory posit that information can be held at different levels of priority in working memory, with information at the highest level of priority in the focus of attention (FoA) and the remaining information in a variously named state of “activated long-term memory” [5] or “region of direct access” [6].

Much of the empirical support for these models comes from tasks using a “retrocuing” procedure that allows for the controlled study of the back-and-forth switching of priority between memory items. In the dual serial retrocuing (DSR) task, 2 items are initially presented as memoranda, followed by a retrocue that designates one the “prioritized memory item” (PMI; equivalent to the “attended memory item” in previous publications) that will be interrogated by the impending probe. The uncued item cannot be dropped from working memory, however, because following the initial memory probe, a second retrocue may indicate (with *p* = 0.5) that this initially uncued item will be tested by the second memory probe. Thus, following the initial retrocue, the uncued item becomes an “unprioritized memory item” (UMI; equivalent to the “unattended memory item” in previous publications) [7].

Functional magnetic resonance imaging (fMRI) and electroencephalography (EEG) studies using the DSR task have suggested that the PMI and UMI may be processed differently. Whereas classification evidence from multivariate pattern analysis (MVPA) for an active delay-period representation of the PMI is robust, particularly in the occipital and temporal regions associated with visual perception and object recognition, evidence for an active representation of the UMI either drops to baseline [8–10] or can only be recovered in rostral, multimodal areas of parietal and frontal cortex [11]. This has led to the suggestion that, for visual stimuli, an elevated level of activation, particularly in the occipital and temporal regions, may be the neural basis of the representation of information in the FoA. However, the neural bases of information held in working memory, but outside the FoA, are less clear.

Synaptic accounts of the retention of unattended information hold that the UMI is maintained in an activity-silent state, potentially through changes in short-term synaptic plasticity in the neural circuits involved in stimulus representation [12–14]. These changes may also occur concurrent with the activity-based representations of the FoA [15]. Consistent with this account, information about the UMI can be recovered by probing the delay period with a pulse of transcranial magnetic stimulation (TMS) [16] or a task-irrelevant visual impulse [17] (c.f. [15,18] for simulations). Another hypothesis is the “cortical specialization” account, whereby all information held in working memory is maintained, possibly in a lower-resolution format, in a specialized circuit in frontal eye fields (FEF) and intraparietal sulcus (IPS) and only the PMI is represented in a high-fidelity representation in occipital cortex [11].

Recently, a third alternative, positing a representational transformation of the UMI, has been proposed. One source of this idea was the results from a dual serial visual search task, in which the pattern of activity representing a search template in object-selective posterior fusiform cortex transitioned to an “opposite” format if a different template was to be searched for prior to the critical template (i.e., when the critical template was the UMI) relative to when it was relevant for the current search [19]. By this representational transformation scheme, both the UMI and the PMI may be encoded simultaneously in posterior cortex, with the format of their neural representation varying with priority status [20].

Although the van Loon and colleagues [19] study provided a new and plausible account for the representation of the UMI, much remains to be explored for this priority-based transformation account. First, it is unclear whether this operation can generalize to different domains of information, for example, low-level visual information. Second, the van Loon and colleagues study [19] observed the opposite pattern only during the search display. Tracking the changes in the neural representations throughout the delay period (with no stimulus on screen) in a typical working memory task would be essential to understand the mechanism of this neural signal. Thus, the current study was designed to assess evidence for a priority-based remapping at 2 novel levels of representation: the item-specific “identity” of oriented-grating stimuli and the trial-unique “context” of each stimulus, operationalized as location. Specifically, we used inverted encoding models (IEMs) to reconstruct the orientation and location of the PMI and of the UMI and tested the remapping hypothesis by comparing the representational formats of the two.

The design, procedures, and hypotheses were preregistered at https://osf.io/g4c3n/.

## Results

**Fig 1:**
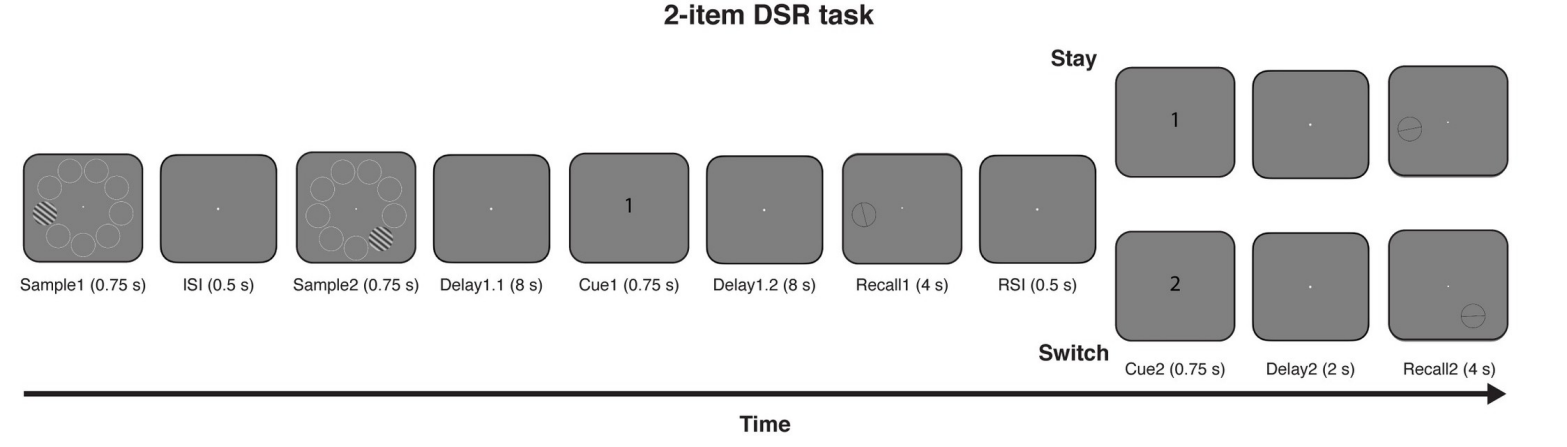
Procedure of the DSR task. Two oriented gratings were presented sequentially, each at one of 9 possible locations (white circles are included for illustration but were not present in the actual experiment). After *Delay1*.*1*, a numeral (*Cue1*) indicated whether the sample item presented first or second would need to be recalled after *Delay 1*.*2*. Recall of the cued item was performed on a recall dial (*Recall1*), then, after a 0.5-s blank interval, a second cue (*Cue2*) indicated whether the sample item presented first or second would need to be recalled after *Delay2*. DSR, dual serial retrocuing.

Participants were scanned with fMRI while performing a DSR task in which 2 gratings (9 possible orientations) were presented serially, each at one of 9 possible locations, and after an initial *Delay1*.*1*, *Cue1* indicated which of the two (first or second) would be tested for recall at the end of the ensuing *Delay1*.*2*. Following *Recall1*, *Cue2* indicated the grating to be tested at the end of *Delay2*, with a 50% of probability for each grating (Stay or Switch; Fig 1). Of primary interest was the neural representation of the sample stimuli during *Delay1*.*2*, when *Cue1* had given one the status of PMI and the other the status of UMI. The neural representation of the orientation and the location of each item were assessed by training 2 IEMs with leave-one-run-out cross-validation. For “PMI-trained IEMs,” the data on each trial were labeled according to the identity of the PMI at the time point of interest, and the converse was true for “UMI-trained” IEMs. Results from the PMI-trained IEMs would be the primary focus of this study because PMI-trained models have been shown to be valid and robust for various brain regions, including occipitotemporal cortex, IPS, and FEF [21–23].

### General behavioral performance

Behavioral responses were analyzed with a 3-factor mixture model [24] that uses maximum likelihood estimation to generate estimates of 1) the probability of responses to the cued item (“responses to target”; *p*T), 2) the probability of responses incorrectly made to the uncued item (“responses to nontarget”; *pN*), and 3) the probability of responses that were guesses, as well as 4) a “concentration” parameter that estimates the precision of nonguess responses. The mixture model estimates for *Recall1* showed a concentration of 21.9 ± 3.6, *p*T of 79.8% ± 1.9%, *p*N of 5.2% ± 1.1%, and guessing of 15.0% ± 1.1%. For *Recall2*, performance on the Stay versus Switch conditions was significantly different in terms of concentration (21.3 ± 3.1 for Stay versus 16.1 ± 2.4 for Switch; *t*[12] = 4.74; *p* < 0.001) and comparable in terms of the other parameters (*p*T: 77.1% ± 2.6% versus 79.0% ± 2.5%; *p*N: 6.8% ± 1.5% versus 7.2% ± 1.4%; and guessing: 15.9% ± 1.5% versus 13.8% ± 1.7%; *t*-values < 0.21; *p*-values > 0.145; for Stay versus Switch, respectively).

### Reconstructions with PMI-trained IEMs

#### Stimulus orientation: Early visual region of interest (ROI)

**Fig 2:**
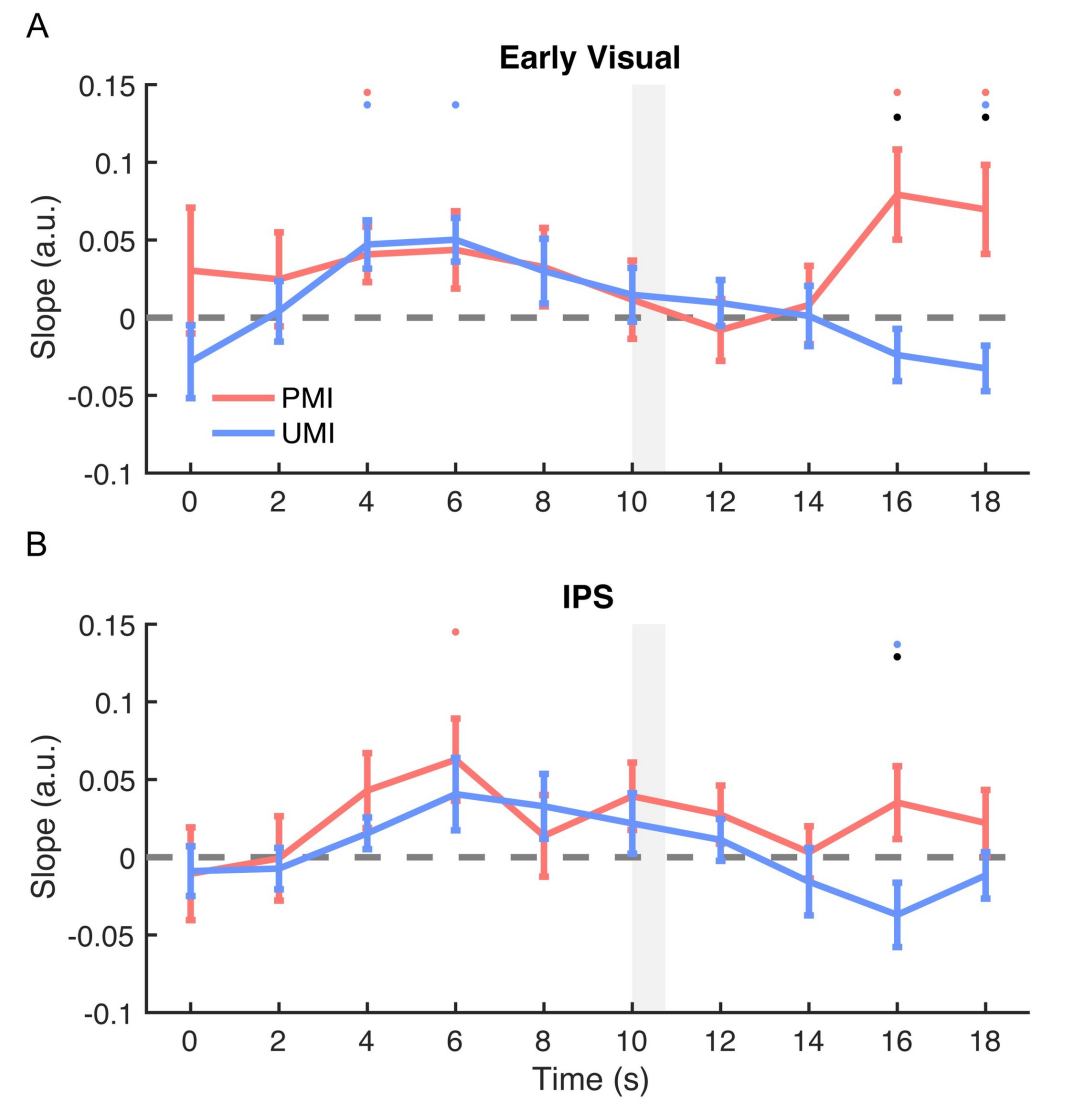
Time course of IEM reconstructions of stimulus orientation. (A) Time course of the slope of orientation reconstructions in early visual ROI. (B) Time course of the slope of orientation reconstructions in IPS ROI. Slopes of the orientation reconstructions of the 2 sample items were plotted as a function of time from the beginning of the trial through the time point concurrent with the end of *Delay1*.*2* and the onset of *Recall1* (0–18 s after trial onset). All results were from PMI-trained IEMs. Red lines represent the PMI, and blue lines represent the UMI. Gray shaded area indicates display of *Cue1* (10–10.75 s). Red, blue, and black dots indicate *p* < 0.05 for significant reconstruction of PMI, significant reconstruction of the UMI, and a significant difference between the two, respectively. All error bars indicate ± 1 SEM. Data are available at osf.io/G4C3N. a.u., arbitrary unit; IEM, inverted encoding model; IPS, intraparietal sulcus; PMI, prioritized memory item; ROI, region of interest; UMI, unprioritized memory item.

For orientation reconstruction in early visual cortex, during portions of *Delay1*.*1*, both the reconstructions of the PMI and the UMI were significant (both positive slopes; *p*-values < 0.020) and were not different from each other (*p*-values > 0.173). The PMI and the UMI started to differ in their reconstruction strength after the onset of *Cue1* (presented at 10 s after trial onset). This difference was significant for 16–18 s after trial onset (*p*-values = 0.013 and 0.025; Fig 2A). These results were consistent with previous findings [8–10] demonstrating a clear priority-based modulation of stimulus representations after retrocuing.

**Fig 3:**
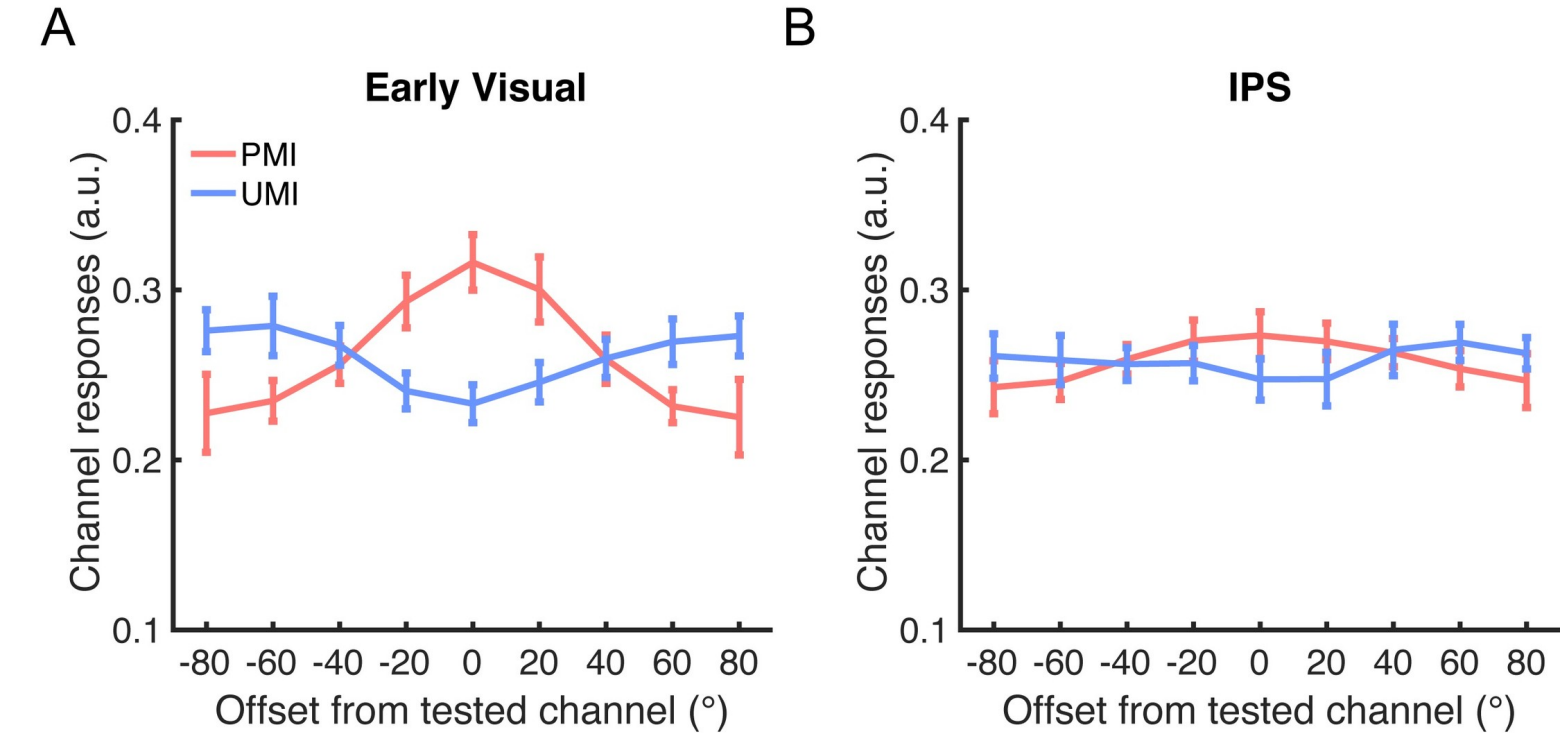
IEM reconstructions of stimulus orientation in *Delay1*.*2*. (A) IEM reconstructions of stimulus orientation during late *Delay1*.*2* (18 s after trial onset) in early visual ROI. (B) IEM reconstructions of stimulus orientation during late *Delay1*.*2* in IPS ROI. All results were from PMI-trained IEMs. Red lines represent the PMI, and blue lines represent the UMI. All error bars indicate ± 1 SEM. Data are available at osf.io/G4C3N. a.u., arbitrary unit; IEM, inverted encoding model; IPS, intraparietal sulcus; PMI, prioritized memory item; ROI, region of interest; UMI, unprioritized memory item.

Critically, at the final time point of *Delay1*.*2* (18 s), the reconstructions of the orientation of the PMI and of the UMI went in opposite directions: there was a significant reconstruction with a positive slope for the PMI (*p* = 0.044) and a significant reconstruction with a negative slope for the UMI (*p* = 0.048; *p*-values corrected for multiple comparisons for this and all the subsequent analyses on time 18 s; Fig 3A). The result for the UMI is particularly noteworthy because it indicates that information about the unprioritized orientation was maintained in an active state in early visual cortex but in a format that differed from its representation when prioritized.

#### Stimulus orientation: IPS ROI

The time course of the reconstructions of orientation in IPS showed no convincing evidence of above-chance reconstruction of either the PMI or the UMI during *Delay1*.*1*, although there was a suggestion of an influence of priority during *Delay1*.*2* that was qualitatively similar to what was observed in the early visual ROI, with the reconstructions of the PMI and the UMI moving in opposite directions following *Cue1*. Reconstruction strengths of the PMI and the UMI differed at 16 s after trial onset (*p* = 0.022), but not at 18 s (*p* = 0.288; Fig 2B). Furthermore, the positive slope of the IEM reconstruction of the PMI was not statistically different from 0 at either 16 or 18 s (*p*-value*s* > 0.128), and that of the UMI was significantly negative at 16 s (*p* = 0.022), but not at 18 s (*p* = 0.638; Fig 3B).

#### Stimulus location: Early visual ROI

**Fig 4:**
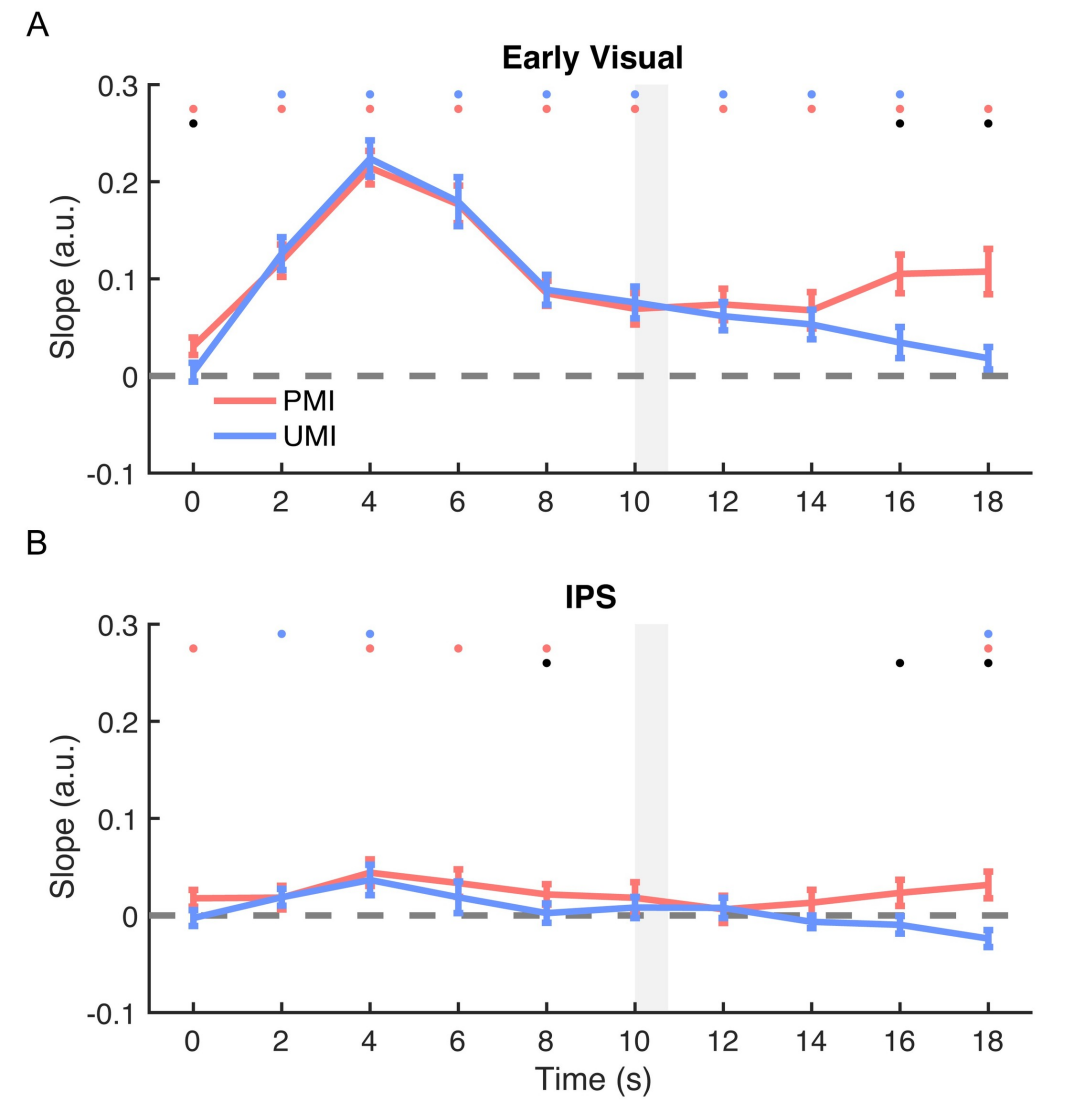
Time course of IEM reconstructions of stimulus location. (A) Time course of the slope of location reconstructions in early visual ROI. (B) Time course of the slope of location reconstructions in IPS ROI. Slopes of the location reconstructions of the 2 sample items were plotted as a function of time from the beginning of the trial through the time point concurrent with the end of *Delay1*.*2* and the onset of *Recall1* (0–18 s after trial onset). All results were from PMI-trained IEMs. Red lines represent the PMI, and blue lines represent the UMI. Gray shaded area indicates display of *Cue1* (10–10.75 s). Red, blue, and black dots indicate *p* < 0.05 for significant reconstruction of PMI, significant reconstruction of the UMI, and a significant difference between the two, respectively. All error bars indicate ± 1 SEM. Data are available at osf.io/G4C3N. a.u., arbitrary unit; IEM, inverted encoding model; IPS, intraparietal sulcus; PMI, prioritized memory item; ROI, region of interest; UMI, unprioritized memory item.

**Fig 5:**
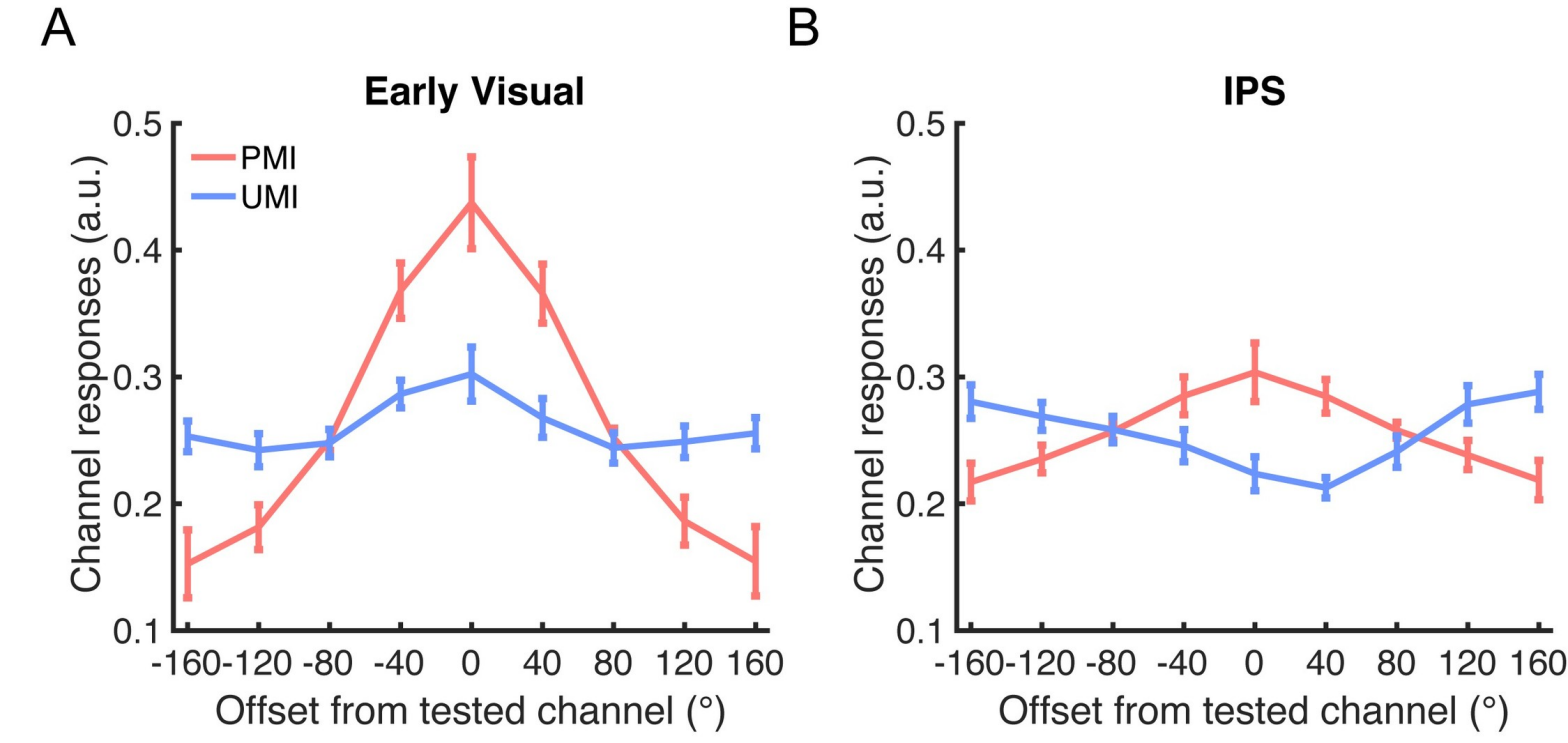
IEM reconstructions of stimulus location in *Delay1*.*2*. (A) IEM reconstructions of stimulus location during late *Delay1*.*2* (18 s after trial onset) in early visual ROI. (B) IEM reconstructions of stimulus location during late *Delay1*.*2* in IPS ROI. All results were from PMI-trained IEMs. Red lines represent the PMI, and blue lines represent the UMI. All error bars indicate ± 1 SEM. Data are available at osf.io/G4C3N. a.u., arbitrary unit; IEM, inverted encoding model; IPS, intraparietal sulcus; PMI, prioritized memory item; ROI, region of interest; UMI, unprioritized memory item.

The location of the PMI could be reconstructed throughout *Delay1*.*1* and *Delay1*.*2* (positive slopes; *p*-values < 0.001; Fig 4A). Reconstruction of the location of the UMI was also robust with a positive slope during *Delay1*.*1* (*p*-values < 0.001), began to decline after the cue, and was no longer different from 0 during the final time point of *Delay1*.*2* (*p* = 0.091, Fig 5A).

#### Stimulus location: IPS ROI

In IPS, the reconstruction time course indicated significant representation of the location of both the PMI and the UMI during portions of *Delay1*.*1* (both positive slopes, *p*-values < 0.026). Priority-based differentiation then developed during *Delay1*.*2*, with the representation of the location of the PMI differing from that of the UMI at 16 s (*p* = 0.027) and 18 s after trial onset (*p* < 0.001; Fig 4B). Critically, at 18 s, the slope of the reconstruction of the location of the PMI was significantly positive (*p* = 0.013), and the slope of the reconstruction of the location of the UMI was significantly negative (*p* = 0.013; Fig 5B). These results indicate an involvement of IPS in maintaining the location context of both prioritized and unprioritized items in working memory.

### Reconstructions with UMI-trained IEMs

#### Stimulus orientation: Early visual ROI

The reconstruction time courses showed above-chance reconstruction of the PMI during portions of *Delay1*.*1* (positive slopes, *p*-values < 0.006), an effect that reversed during *Delay1*.*2* (negative slopes, *p*-values < 0.001). At 16 s of *Delay1*.*2*, the difference between the UMI and the PMI was significant (*p* = 0.032; S1A Fig). At 18 s, the reconstruction of the UMI was not successful (*p* = 0.494), but the reconstruction of the PMI was successful (negative slope, *p* < 0.0001; S2A Fig).

#### Stimulus orientation: IPS ROI

The time course showed above-chance reconstructions for both the PMI and the UMI during portions of *Delay1*.*1* and then only for the UMI during early *Delay1*.*2* (positive slopes, *p*-values < 0.048; S1B Fig). However, neither reconstruction was significant during late *Delay1*.*2* (*p*-values > 0.143, S2B Fig).

#### Stimulus location: Early visual ROI

The reconstruction time courses showed significant reconstructions for the PMI and the UMI throughout *Delay1*.*1* and above-chance reconstructions for the two for part of early *Delay1*.*2* (both positive slopes, *p*-values < 0.016; S3A Fig). During late *Delay1*.*2*, neither location could be reconstructed (*p*-values = 0.124; S4A Fig).

#### Stimulus location: IPS ROI

There was above-chance reconstruction for the PMI and for the UMI for portions of *Delay1*.*1* (positive slopes, *p*-values < 0.012), but not for *Delay1*.*2*, although a difference between the PMI and the UMI emerged at 18 s during *Delay1*.*2* (*p* = 0.045; S3B Fig). When focusing on 18 s, neither the UMI nor the PMI location could be reconstructed in IPS (*p*-values = 0.126 and 0.218; S4B Fig), although the pattern was consistent with the PMI-trained IEM results, with a positive trending slope for the UMI and a negative trending slope for the PMI.

### Temporal generalization of reconstructions

Finally, it is worthy of note that temporal generalization analysis demonstrated failed generalization between the encoding and the maintenance periods for both orientation (S5 Fig) and location (S6 Fig), suggesting a dynamic change in neural code from the encoding to different maintenance periods in the DSR task (full statistical results can be found in S1–S8 Tables).

## Discussion

What neural mechanism underlies the maintenance of working memory content with different priorities? The present results suggest that, when deprioritized, the neural representations of an item’s identity and of its context undergo a transformation that we characterize as priority-based remapping. With fMRI, a neural code refers to the systematic set of mappings between unique stimulus values and unique patterns of neural activity, the property that supports multivariate decoding and encoding. The present results, for example, indicate that 9 values of stimulus orientation map to 9 different high-dimensional patterns of activity within early visual cortex. Furthermore, they suggest that when a stimulus transitions to an unprioritized status, this set of mappings rotates such that the individual mappings between stimulus values and neural patterns are now different, but the distance (in orientation) between neural patterns is preserved. Thus, although the item’s neural representation has changed, the code underlying the representation of orientation has not changed. For this reason, we characterize the priority-based transformations reported here as examples of remapping, not of recoding. This remapping account is also consistent with the findings of van Loon and colleagues [19], who observed that a classifier trained on a stimulus category when it was the PMI could also decode that category when it was the UMI, even though the pattern of activity of the UMI projected into an opposite region of multidimensional scaling space relative to the PMI. The characteristics underlying priority-based remapping may account for some of the mixed results in previous work on the effects of retrocuing.

### Stimulus identity

In this study, IEM of delay-period activity indicated that early visual cortex represents the orientation of grating stimuli when they are being held in the FoA, an observation that is broadly consistent with previous neuroimaging studies of working memory for stimuli defined by low-level visual features (for example, [22,25–27]). When a retrocue designated an item a UMI, IEM indicated that its representation in early visual cortex underwent a transformation that corresponded to a rotation of 90° relative to how it was represented in the FoA. By extension, the present results also lend support to the interpretation of the priority-based transformation reported by van Loon and colleagues [19]. Because they observed a transformation of the posterior inferior temporal representation of objects in their study (cows, dressers, ice skates) to an “opposite” pattern in multidimensional scaling space, the general principle underlying priority-based remapping may be one of transforming the representation of deprioritized information into a format that is complementary to its representation when in the FoA, effectively maximizing the difference between an item when it is a UMI versus when it is a PMI. Furthermore, although the design of the present study did not allow us to assess stimulus representation during *Delay2*, results from a different study that used a similar procedure suggest that on “switch” trials, the representation of the previously deprioritized item rotates back to its PMI format [28].

### Stimulus context

The binding of information about a stimulus to its trial-unique context is fundamental to what it means to hold information in working memory [29,30]. In the present study, the location at which the items held in working memory had been presented (i.e., their context) could be successfully reconstructed in IPS, and the format of these representations also displayed priority-based remapping. This effect, as measured by IEM, corresponded to a rotation of the mnemonic representation of an item’s location by 180° when it was a UMI relative to when it was in the FoA.

These results are consistent with the idea that a parietal priority map tracks the location context of all items held in visual working memory and that, similar to the neural representation of stimulus identity, the priority-based representational transformation of location context is also implemented via rotational remapping. Interestingly, in the present study, location context was not needed for task performance because items were cued according to order of presentation. These results are consistent with previous reports of an automatic encoding of location information [31], suggesting that the binding between location and identity is an intrinsic process in working memory for objects.

### What brain areas support priority-based remapping?

Noteworthy in our results is the fact that the priority-based remapping of stimulus orientation was observed in early visual cortex, but not in IPS, whereas the priority-based remapping of stimulus location was observed in IPS, but not in early visual cortex. Considering orientation, we cannot rule out the possibility that our study may have simply lacked the power to detect the remapping of orientation in IPS. Already in *Delay1*.*1*, when both items were in the FoA, there was only weak evidence for the representation of orientation in IPS, and we know from previous work that successful decoding of low-level visual features is less robust in IPS relative to early visual cortex, particularly in conditions, like high memory load, when stimulus information is weaker (for example, [32]). This low-sensitivity account seems much less plausible, however, for the representation of location, which was robust in early visual cortex for the PMI. Two other factors that may be important for understanding our results are domain specificity and task-specific function.

A domain-specificity account would predict that priority-based remapping is predominantly engaged in regions that are necessary for representing the visual feature in question. From this perspective, our results may have been predictable from the facts that damage to occipital areas, but not IPS, can produce apperceptive agnosia [33], and that damage to IPS, but not to early visual areas, can produce disordered spatial cognition [34]. It is noteworthy, in this regard, that van Loon and colleagues [19] observed priority-based remapping of category representations in fusiform gyrus. A functional account would emphasize the content/context distinction. For tasks for which stimulus location serves as context, the representation of location in IPS might undergo priority-based remapping because of this region’s role in context binding (for example, [32]). From this perspective, despite the strong representation of retinotopy in early visual cortex, the absence of a functional role for this brain area in context binding may explain the absence of evidence for remapping of the representation of location. It will be interesting, in future work, to pit these 2 accounts against each other with a task in which stimulus location is the to-be-remembered content and orientation the context that is used for cuing priority.

### Comparison with previous results

Why did the present study and that of van Loon and colleagues [19] find evidence for active representation of the UMI in stimulus-representing cortex when many previous attempts with the DSR task have been unsuccessful? One important factor is how the classifier/encoding model is trained for probing the representation of the UMI. In previous studies, researchers either used a separate 1-item task to train the classifier [8–10] or used a regression model based on the UMI [11]. In the present study and in van Loon and colleagues [19], it was a decoder/encoder based on the PMI that found evidence for active neural representation of the UMI. Decoders trained on a different task or on the UMI may not be able to discriminate representations that are rotations of the PMI.

The trend in the present study—that results with UMI-trained models were less robust than those with PMI-trained models—is also consistent with previous work. For example, Christophel and colleagues [11] could not decode UMI-related information in an ROI comprising V1–V4 (although decoder performance came closer to statistical significance when the analysis was restricted to V3 and V4). In the study from van Loon and colleagues [19], although decoding of the UMI using a UMI-trained classifier was statistically significant, the decoding accuracy was markedly lower in comparison with using a PMI-trained classifier. Together, the 3 sets of results are consistent with the idea that UMI-trained models/classifiers are not as effective as PMI-trained models/classifiers.

A notable inconsistency between the present results and those from a previous study that used the DSR technique [11] is the absence, in this study, of evidence for an active representation of the UMI in IPS, whether with PMI- or UMI-trained IEMs. One factor, mentioned above, is that in our experience, successful decoding of low-level visual features is less robust in IPS relative to early visual cortex (for example, [32]). Perhaps relatedly, it has been suggested that the representation of stimulus identity in IPS is lower resolution and weaker than in early visual cortex (c.f. [11]), factors that could have influenced our results.

### Possible alternative accounts

Because the prioritization-related changes that we have documented here manifest as a “flipping” of IEM reconstructions, there are at least 2 alternatives to priority-based remapping that need to be considered: undershoot and inhibition. Undershoot can occur when the drive on activity in a region is removed, such as for early visual cortex with the offset of visual stimulation. In the present study, an undershoot account would hold that activity in voxels representing the UMI return to baseline when the retrocue triggers the shift of attention to the PMI. Importantly, because undershoot is a passive phenomenon, one would expect it to be present for all conditions. In the present results, however, the effects were specific to domain and to brain region: they were observed in early visual cortex for the representation of orientation, but not of location, and they were observed in IPS for location, but not for orientation. Furthermore, because in early visual cortex, the *Delay1*.*1* representation of location was greater in amplitude than was that of orientation, any undershoot in that region would be expected to be greater for location than for orientation.

A second alternative account of the prioritization-related changes reported here is stimulus-specific inhibition or suppression. By this account, if the signal intensity in voxels driving the orientation channel that the UMI is centered on is suppressed, spontaneous activity in voxels that drive off-orientation channels could cause the inversion in channel responses observed in the IEM results. Further computational modeling may be useful for differentiating between different accounts.

It is also important to note, as we consider alternative accounts, that priority-based remapping of active representations is not incompatible with the previously proposed, activity-silent mechanism of retaining the UMI. Because remapping of the active representations of stimulus information was only evident later in the delay period, it is possible that remapping also occurred at the synaptic level before the representation became reactivated. It is also possible that remapping occurred at the activity level with the activity-silent representation unchanged. Therefore, several different mechanisms may work together to support the representation of priority in working memory.

### Priority-based remapping in visual working memory

The proposed mechanism of priority-based remapping can be considered at several levels of description. At the level of its neural implementation, priority-based remapping may be accomplished via a systematic reweighting of the weights that map from neuron space into the lower-dimensional space of population-level representation. This is reminiscent of the ordinal reversal of voxel signal intensities between encoding and retention that has been reported in primary auditory cortex for auditory working memory [35]. It is also reminiscent of the rotational dynamics described in mouse auditory cortex by Libby and Buschman [36], a result of a subset of the neurons representing a memory dynamically inverting their selectivity.

At the level of control, priority-based remapping has been modeled as a consequence of competition between prefrontal pointers that activate their corresponding perceptual representations with different levels of priority [18,37]. Whether the effects that we observed in parietal cortex may also reflect the operation of a source of the priority-based control of information held in working memory, as implemented via the priority-sensitive representation of stimulus context, is an important question for future research.

Finally, at a theoretical level, why should the features of a stimulus be represented in rotated formats when the stimulus is a UMI versus when it is a PMI? One possible explanation is that such remapping may be an effective way to keep information in an active, accessible state while also accomplishing the simultaneous goals of protecting it from degradation and minimizing the likelihood that it will interfere with the real-time guidance of behavior. Similar ideas have been proposed, for example, as a basis for retaining remembered information in noisy neural networks [37] or the projection of active representations into a null space [38] as a means of maintaining multiple representations of goal states [39].

To conclude, with multivariate IEMs, we observed and quantified changes in the neural representation of stimulus information as a function of attentional prioritization. In early visual cortex, the representation of the orientation of a sample transformed into an opposite pattern when it transitioned from a PMI to a UMI. In IPS, the same was true for the representation of the location at which that item had been presented. These results suggest a mechanism for a priority-based remapping of information when it is held in working memory but outside the FoA.

## Methods

### Ethics statement

This study was approved by the University of Wisconsin-Madison Health Sciences Institutional Review Board (2017–0344) and was conducted according to the principles of the Declaration of Helsinki. Participants provided written informed consent prior to participation.

### Participants

An a priori power analysis based on the effect sizes found in a previous experiment [20] indicated that a target sample size of 13 subjects was needed to detect the smallest of the effects predicted by our hypotheses. A total of 14 individuals participated in the study (3 male, average age 21.1 ± 4.5 years), with one subsequently excluded because of excessive head movement. All were recruited from the University of Wisconsin-Madison community. All had normal or corrected-to-normal vision and were neurologically healthy. All participants were monetarily compensated for their participation.

### Stimuli and procedure

All stimuli were created and presented using MATLAB (The MathWorks, Natick, MA, USA) and Psychtoolbox 3 extensions [40,41] on a 60-Hz Avotec Silent Vision 6011 projector (Avotec, Stuart, FL, USA) and viewed through a coil-mounted mirror in the MRI scanner. A trackball response pad (Current Designs, Philadelphia, PA, USA) was employed to record the behavioral responses.

Participants performed a DSR task in the scanner. A white fixation dot was presented at the center of the screen throughout the experiment. On each trial, participants first viewed 2 sample stimuli (sinusoidal gratings: radius = 5°; contrast = 0.6; spatial frequency = 0.5 cycles/°; phase angle randomized between 0° and 180°) presented sequentially on the screen (0.75 s exposure or each, separated by an ISI of 0.5 s). The orientation of each sample was selected independently from a fixed set of 9 values, spaced by 20° and with a jitter between 0°–3° added to each, and the location of each was selected independently from a fixed set of 9 locations, each centered on an imaginary circle with radius of 8° from central fixation and each spaced 40° distant from the nearest locations. Offset of the second sample was followed by a delay period (*Delay1*.*1*, of 8 s), then a retrocue (*Cue1*; central presentation for 0.75 s) specifying whether the sample presented first or second (“1” or “2”) would need to be recalled at the end of *Delay1*.*2* (8 s). Recall was prompted by an orientation wheel appearing at the same location as the cued sample, and participants had been trained to use it to reproduce the cued orientation within a 4-s response window (*Recall1*). 0.5 s after the end of *Recall1*, a second retrocue (*Cue2*, central presentation for 0.75 s), indicated which sample (“1” or “2”) need to be recalled at the end of *Delay2* (2 s). On 50% of trials, *Cue2* cued the same item that had been cued by *Cue1* (a “Stay” cue), and for the remaining 50%, it cued the previously uncued sample (a “Switch” cue). *Recall2* (same procedure as *Recall1*) was followed by an intertrial interval that varied randomly between 6, 8, and 10 seconds (Fig 1). During *Delay1*.*2*, the cued sample was termed the PMI and the uncued sample the UMI.

All participants completed 2 fMRI scanning sessions. For the first 4 participants, the first scanning session comprised 12 blocks of 12 trials each, and a second scanning session comprised thirteen 12-trial blocks for a total of 300 trials. Each scanned block lasted 464 s. The selection of stimulus location and orientation were independent, such that the orientation of the second sample matched that of the first on approximately one-ninth of the trials, and the location of the second sample matched that of the first on approximately one-ninth of the trials. For the remaining participants, we fully counterbalanced the conditions. To fully cross stimulus orientation, stimulus location, *Cue1*, and *Cue2*, 324 trials were required. To achieve this, participants 5–13 performed thirteen 12-trial runs during the first scanning session and fourteen 12-trial runs during the second scanning session. Each run consisted of 12 trials, resulting in a run length of 464 s. Before the first session, each participant completed 2 blocks of practice trials (12 trials per block) outside of the scanner and another block of practice within the scanner before the fMRI scanning began. During the scan, eye position was monitored and recorded using the Avotec RE-5700 eye-tracking system (Avotec).

This design was modified from a previous study [42] that found that in early visual cortex, the IEM reconstruction of a stimulus’ orientation when a UMI was opposite to its reconstruction when a PMI, and in IPS, the IEM reconstruction of its location when a UMI was opposite to its reconstruction when a PMI. One complication for the representational transformation interpretation of those results, however, was that there existed in both a negative correlation between the identities of the PMI and UMI: both tasks were designed such that the PMI and UMI could never be the same on a single trial. This negative correlation could conceivably produce negative IEM reconstruction for analytic reasons. In the present study, the orientation and location of each trial’s samples were selected independently. This aspect of the design, however, meant that retrocuing in terms of an item’s location would be ambiguous on trials when the 2 samples appeared at the same location, and so sequential presentation and cuing by order were instituted. Note that although this aspect of the procedure meant that stimulus location was not task-critical (for example, one could ignore or forget where the samples had appeared and still succeed at the task), we expected that participants would nonetheless represent sample location in working memory. For example, previous work has indicated that stimulus location is encoded robustly during working memory for the orientation of individually presented stimuli [23,31].

### Behavioral analysis

We analyzed behavioral responses with a 3-factor mixture model [24] that uses maximum likelihood estimation to generate estimates of 1) the probability of responses based on a representation of the probed item (“responses to target”), 2) the probability of responses incorrectly based on a representation of the unprobed item (i.e., “misbinding” or “swap” errors (“responses to nontarget”), and 3) the probability of responses that were guesses not based on either memory item, as well as 4) a “concentration” parameter that estimates the precision of nonguess responses. Conceptually, the concentration parameter is similar to a model-free measure of the precision of responses that is computed as the inverse of the standard deviation of the distribution of responses.

### fMRI data acquisition

Whole-brain images were acquired using a 3 Tesla GE MR scanner (Discovery MR750; GE Healthcare, Chicago, IL, USA) at the Lane Neuroimaging Laboratory at the University of Wisconsin-Madison HealthEmotions Research Institute (Department of Psychiatry). Functional imaging was conducted using a gradient-echo echo-planar sequence (2 s repetition time [TR], 22 ms echo time [TE], 60° flip angle) within a 64 × 64 matrix (42 axial slices, 3 mm isotropic). A high-resolution T1 image was also acquired for each session with a fast, spoiled gradient-recalled-echo sequence (8.2 ms TR, 3.2 ms TE, 12° flip angle, 176 axial slices, 256 × 256 in- plane, 1.0 mm isotropic).

### fMRI data preprocessing

Functional MRI data were preprocessed using AFNI (http://afni.nimh.nih.gov) [43]. The data were first registered to the final volume of each scan and then to anatomical images of the first scan session. Six nuisance regressors were included in GLMs to account for head motion artifacts in 6 different directions. The data were then motion corrected, detrended, and z-score normalized within each run.

### fMRI ROI definition

The ROIs were created with a conjunction of anatomically and functionally defined voxels. We first created anatomical ROIs by extracting masks from the probabilistic atlas of Wang and colleagues [44] and warping them to each subject’s structural scan in native space to create 2 regional masks, defining an early visual ROI as V1–V2 (merged, both hemispheres) and an IPS ROI as IPS0–5 (merged, both hemispheres). To identify task-related activity, we solved a general linear model (GLM) with AFNI, modeling each epoch of the task with 6 boxcar regressors—*Sample* (2 s), *Delay1*.*1* (8 s), *Delay1*.*2* (8 s), *Recall1* (4 s), *Delay2* (2 s), and *Recall2* (4 s)—convolved with a canonical hemodynamic response function and also including covariates to control for motion. We then created an anatomically constrained functional ROI for bilateral early visual cortex by selecting the 500 voxels inside the early visual anatomical ROI with the strongest loading on the *Sample* regressor and for bilateral IPS by selecting the 500 voxels inside the IPS anatomical ROI with highest loading on the *Delay1*.*2* regressor.

### Multivariate IEM

#### Mathematics

All IEM analyses were performed using custom functions in MATLAB. The IEM assumes that the responses of each voxel can be characterized by a small number of hypothesized tuning channels. The numbers of orientation and location tuning channels were both 9. Following previous work [21,45], the idealized feature tuning curve of each channel was defined as a half-wave–rectified sinusoid raised to the eighth power for both orientation and location.

For the IEM, we first computed the weight matrix (*W*) that projects the hypothesized channel responses (*C*_*1*_) to actual measured fMRI signals in the training data set (*B*_*1*_) and then extracted the estimated channel responses (${\hat{C}}_{2}$) for the test data set (*B*_*2*_) using this weight matrix. The relationship between the training data set (*B*_*1*_, *v* × *n*, *v*: the number of voxels in the ROI; *n*: the number of repeated measurements) and the channel responses (*C*_*1*_, *k* × *n*, *k*: the number of orientations/locations) was characterized by

$$B_{1}=WC_{1},$$

where *W* was the weight matrix (*v* × *k*).

Therefore, the least-squared estimate of the weight matrix ($\hat{W}$) was calculated using linear regression:

$$\hat{W}=B_{1}C_{1}^{T}{\left(C_{1}C_{1}^{T}\right)}^{-1}.$$

The channel responses (${\hat{C}}_{2}$) for the test data set (*B*_*2*_) was then estimated using the weight matrix ($\hat{W}$):

$${\hat{C}}_{2}={\left({\hat{W}}^{T}\hat{W}\right)}^{-1}{\hat{W}}^{T}B_{2}.$$

### The logic behind inferring neural codes from patterns of IEM training and testing

We understand a neural code to refer to the systematic set of mappings between unique stimulus values and unique patterns of neural activity. For example, 9 values of stimulus orientation might map to 9 different high-dimensional patterns of activity within an ROI. One way in which neural coding in posterior cortex could vary with an item’s priority status would be if this set of mappings were to rotate such that the individual mappings between these stimulus values and these neural patterns are now different, but the distance between neural patterns is preserved. In other words, although each item’s neural representation has changed, the neural code has not changed. Therefore, we would consider such a transformation to be an example of remapping (of stimulus value to neural pattern), not of recoding. Such remapping would be consistent with the findings of van Loon and colleagues [19], who observed that a classifier trained on a stimulus category when it was the PMI could also decode that category when it was the UMI, even though the pattern of activity of the UMI projected into an opposite region of multidimensional scaling space relative to the PMI. Another way in which neural coding could vary with priority status would be if an altogether different set of mappings between the 9 stimulus values and 9 new patterns of activity were established. This would correspond to recruiting a different neural code. Under such a recoding scenario, cross-condition classification, such as described by van Loon and colleagues [19], would fail.

In the present study, neural codes were operationalized by IEMs, and 4 different patterns of results were anticipated as possible outcomes. We illustrate them here with reference to stimulus orientation. The most straightforward scenario would be when an IEM trained on the orientation of stimuli when they are PMIs can successfully reconstruct the orientation of the same stimuli when they are UMIs. This would be interpreted as evidence that stimulus orientation is represented in the same neural code regardless of priority status. A second pattern could be that a PMI-trained IEM can successfully reconstruct the orientation of UMIs but does so in such a way that the reconstructed orientation is systematically shifted by a constant amount. For example, a stimulus with 0° orientation reconstructs as 90° when it is a UMI, and a stimulus with 30° orientation reconstructs as 120° when it is a UMI. This would be interpreted as evidence for a rotational remapping within the same neural code because it would correspond to the process described in the previous paragraph. A third pattern could be that a PMI-trained IEM fails to reconstruct the UMI, but the UMI can be reconstructed with a different IEM (for example, with a UMI-trained IEM). This would be interpreted as evidence that the same stimulus information is represented in different neural codes depending on priority status. The final possible outcome that we considered would be the failure to reconstruct the orientation of the UMI with any IEM, which would amount to a failure to find evidence for an active representation of the UMI (in the context of all the possible IEMs that have been trained).

### Analysis plan

We used a leave-one-run-out cross-validation procedure to train and test IEMs, building weight matrices with signals from a time point of interest from the trials from all but one of the runs, then tested on the signals from that same time point from the trials in the held-out run. This procedure was iterated such that each run was tested using a separate training data set. The estimated channel outputs obtained after each iteration were shifted to a common center, with 0° corresponding to the tested feature (orientation/location) channel.

Specifically, for “PMI-trained IEMs,” the data on each trial were labeled according to the identity of the PMI at the time point of interest, such that the model learned each of the 9 possible values of stimulus orientation. Importantly, because the orientation of the UMI was random relative to the PMI on each of these trials, a PMI-trained IEM could not learn any information about the UMI. For “UMI-trained” IEMs, the data from the same time points were labeled according to the identity of the UMI. For PMI-trained IEMs, the IEMs were tested on data labeled according to the identity of the PMI as well as on data labeled according to the identity of the UMI. When tested with UMI-labeled data, reconstructions from this PMI-trained IEM would index the extent to which the representational format of the UMI was similar to that of the PMI. Results from the PMI-trained IEMs would be the primary focus of this study because PMI-trained models have been shown to be valid and robust for various brain regions, including occipitotemporal cortex, IPS, and FEF [21–23]. For UMI-trained IEMs, the IEM was trained on data labeled according to the identity of the UMI and tested on data labeled according to the identity of the both the PMI and the UMI.

For each IEM, we first examined the time course of reconstructions from trial onset to the onset of *Recall1* (i.e., from 0 to 18 s after trial onset), which demonstrated how representations of the PMI and of the UMI evolved before and after *Cue1* (i.e., during *Delay1*.*1* and *Delay1*.*2*). Moreover, because we were interested in the delay period after the retrocue onset (*Delay1*.*2*), we focused on 18 s after trial onset (i.e., 7.25 s after *Cue1* offset) for statistical comparisons in order to maximize the likelihood that our analyses would capture the effect of the retrocue while taking into account the hemodynamic lag in the BOLD signal. All the IEMs were estimated for orientations and locations separately. Temporal generalization analyses were conducted by training and testing the IEM on every time point to examine whether the neural code at a specific time point could be successfully generalized to another.

### Statistical analyses

To characterize the strength of each reconstruction, we collapsed over the channel responses on both sides of the tested channel, averaged them, and calculated the slope of each collapsed reconstruction using linear regression [31,46]. A larger positive slope indicates stronger positive representation, and a larger negative slope indicates stronger negative representation. We used a bootstrapping procedure [21] to characterize the significance of the slopes. For each IEM/ROI, 10 orientation/location reconstructions were randomly sampled with replacement from the reconstruction pool of 13 participants and averaged. This procedure was repeated 10,000 times, resulting in 10,000 average orientation/location reconstructions for each IEM/ROI and, correspondingly, 10,000 slopes. To obtain a two-tailed measure of the *p*-values, the probabilities of obtaining a positive (*p*_pos_; reconstruction peaking at the tested channel) or negative (*p*_neg_) slope among the 10,000 slopes were calculated separately, and the *p*-values of the bootstrapping tests were calculated using the following equation:

$$p=2*min(p_{pos},p_{neg}).$$

To characterize the difference between 2 slopes, we first calculated the difference between 2 bootstrapped slopes 10,000 times, which generated 10,000 slope differences. The significance of the slope difference was then calculated using the same two-tailed method as above. All the *p*-values for the analysis at 18 s were corrected for multiple comparisons across conditions and ROIs; all the *p*-values for the time course analysis remained uncorrected.

## Supporting information

S1 Fig Time course of IEM reconstructions of stimulus orientation using UMI-trained IEMs. (A) Time course of the slope of orientation reconstructions in early visual ROI. (B) Time course of the slope of orientation reconstructions in IPS ROI. Slopes of the orientation reconstructions of the 2 sample items were plotted as a function of time from the beginning of the trial through the time point concurrent with the end of *Delay1*.*2* and the onset of *Recall1* (0–18 s after trial onset). All results were from UMI-trained IEMs. Red lines represent the PMI, and blue lines represent the UMI. Gray shaded area indicates display of *Cue1* (10–10.75 s). Red, blue, and black dots indicate *p* < 0.05 for significant reconstruction of PMI, significant reconstruction of the UMI, and a significant difference between the two, respectively. All error bars indicate ± 1 SEM. Data are available at osf.io/G4C3N. IEM, inverted encoding model; IPS, intraparietal sulcus; PMI, prioritized memory item; ROI, region of interest; UMI, unprioritized memory item.
(TIF)

S2 Fig IEM reconstructions of stimulus orientation in *Delay1*.*2* using UMI-trained IEMs. (A) IEM reconstructions of stimulus orientation during late *Delay1*.*2* (18 s after trial onset) in early visual ROI. (B) IEM reconstructions of stimulus orientation during late *Delay1*.*2* in IPS ROI. All results were from UMI-trained IEMs. Red lines represent the PMI, and blue lines represent the UMI. All error bars indicate ± 1 SEM. Data are available at osf.io/G4C3N. IEM, inverted encoding model; IPS, intraparietal sulcus; PMI, prioritized memory item; ROI, region of interest; UMI, unprioritized memory item.
(TIF)

S3 Fig Time course of IEM reconstructions of stimulus location using UMI-trained IEMs. (A) Time course of the slope of location reconstructions in early visual ROI. (B) Time course of the slope of location reconstructions in IPS ROI. Slopes of the location reconstructions of the 2 sample items were plotted as a function of time from the beginning of the trial through the time point concurrent with the end of *Delay1*.*2* and the onset of *Recall1* (0–18 s after trial onset). All results were from UMI-trained IEMs. Red lines represent the PMI, and blue lines represent the UMI. Gray shaded area indicates display of *Cue1* (10–10.75 s). Red, blue, and black dots indicate *p* < 0.05 for significant reconstruction of PMI, significant reconstruction of the UMI, and a significant difference between the two, respectively. All error bars indicate ± 1 SEM. Data are available at osf.io/G4C3N. IEM, inverted encoding model; IPS, intraparietal sulcus; PMI, prioritized memory item; ROI, region of interest; UMI, unprioritized memory item.
(TIF)

S4 Fig IEM reconstructions of stimulus location in Delay1.2 using UMI-trained IEMs. (A) IEM reconstructions of stimulus location during late *Delay1*.*2* (18 s after trial onset) in early visual ROI. (B) IEM reconstructions of stimulus location during late *Delay1*.*2* in IPS ROI. All results were from UMI-trained IEMs. Red lines represent the PMI, and blue lines represent the UMI. All error bars indicate ± 1 SEM. Data are available at osf.io/G4C3N. IEM, inverted encoding model; IPS, intraparietal sulcus; PMI, prioritized memory item; ROI, region of interest; UMI, unprioritized memory item.
(TIF)

S5 Fig Time-point-by-time-point temporal generalization of orientation. Temporal generalization of orientation reconstructions, in early visual and IPS ROIs, for PMIs and UMIs using PMI-trained IEMs. Strength of reconstructions are indicated by the slope of reconstructions. The x- and y-axes show the tested and training time points, respectively. Data are available at osf.io/G4C3N. IEM, inverted encoding model; IPS, intraparietal sulcus; PMI, prioritized memory item; ROI, region of interest; UMI, unprioritized memory item.
(TIF)

S6 Fig Time-point-by-time-point temporal generalization of location. Temporal generalization of location reconstructions, in early visual and IPS ROIs, for PMIs and UMIs using PMI-trained IEMs. Strength of reconstructions are indicated by the slope of reconstructions. The x- and y-axes show the tested and training time points, respectively. Data are available at osf.io/G4C3N. IEM, inverted encoding model; IPS, intraparietal sulcus; PMI, prioritized memory item; ROI, region of interest; UMI, unprioritized memory item.
(TIF)

S1 Table *p*-Values (uncorrected) of time-point-by-time-point temporal generalization of orientation reconstructions of PMI in early visual cortex. PMI, prioritized memory item.
(XLSX)

S2 Table *p*-Values (uncorrected) of time-point-by-time-point temporal generalization of orientation reconstructions of UMI in early visual cortex. UMI, unprioritized memory item.
(XLSX)

S3 Table *p*-Values (uncorrected) of time-point-by-time-point temporal generalization of orientation reconstructions of PMI in IPS. IPS, intraparietal sulcus; PMI, prioritized memory item.
(XLSX)

S4 Table *p*-Values (uncorrected) of time-point-by-time-point temporal generalization of orientation reconstructions of UMI in IPS. IPS, intraparietal sulcus; UMI, unprioritized memory item.
(XLSX)

S5 Table *p*-Values of time-point-by-time-point temporal generalization of location reconstructions of PMI in early visual cortex. PMI, prioritized memory item.
(XLSX)

S6 Table *p*-Values (uncorrected) of time-point-by-time-point temporal generalization of location reconstructions of UMI in early visual cortex. UMI, unprioritized memory item.
(XLSX)

S7 Table *p*-Values (uncorrected) of time-point-by-time-point temporal generalization of location reconstructions of PMI in IPS. IPS, intraparietal sulcus; PMI, prioritized memory item.
(XLSX)

S8 Table *p*-Values (uncorrected) of time-point-by-time-point temporal generalization of location reconstructions of UMI in IPS. IPS, intraparietal sulcus; UMI, unprioritized memory item.
(XLSX)

## Abbreviations

DSR: dual serial retrocuing
EEG: electroencephalography
FEF: frontal eye fields
fMRI: functional magnetic resonance imaging
FoA: focus of attention
GLM: general linear model
IEM: inverted encoding model
IPS: intraparietal sulcus
MVPA: multivariate pattern analysis
PMI: prioritized memory item
ROI: region of interest
TE: echo time
TMS: transcranial magnetic stimulation
TR: repetition time
UMI: unprioritized memory item
