# Supplementary figures and images for: Different states of priority recruit different neural representations in visual working memory
Source: PLoS Biol. 2020 Jun 29;18(6):e3000769. doi: 10.1371/journal.pbio.3000769 (PMC7351225; doi:10.1371/journal.pbio.3000769)

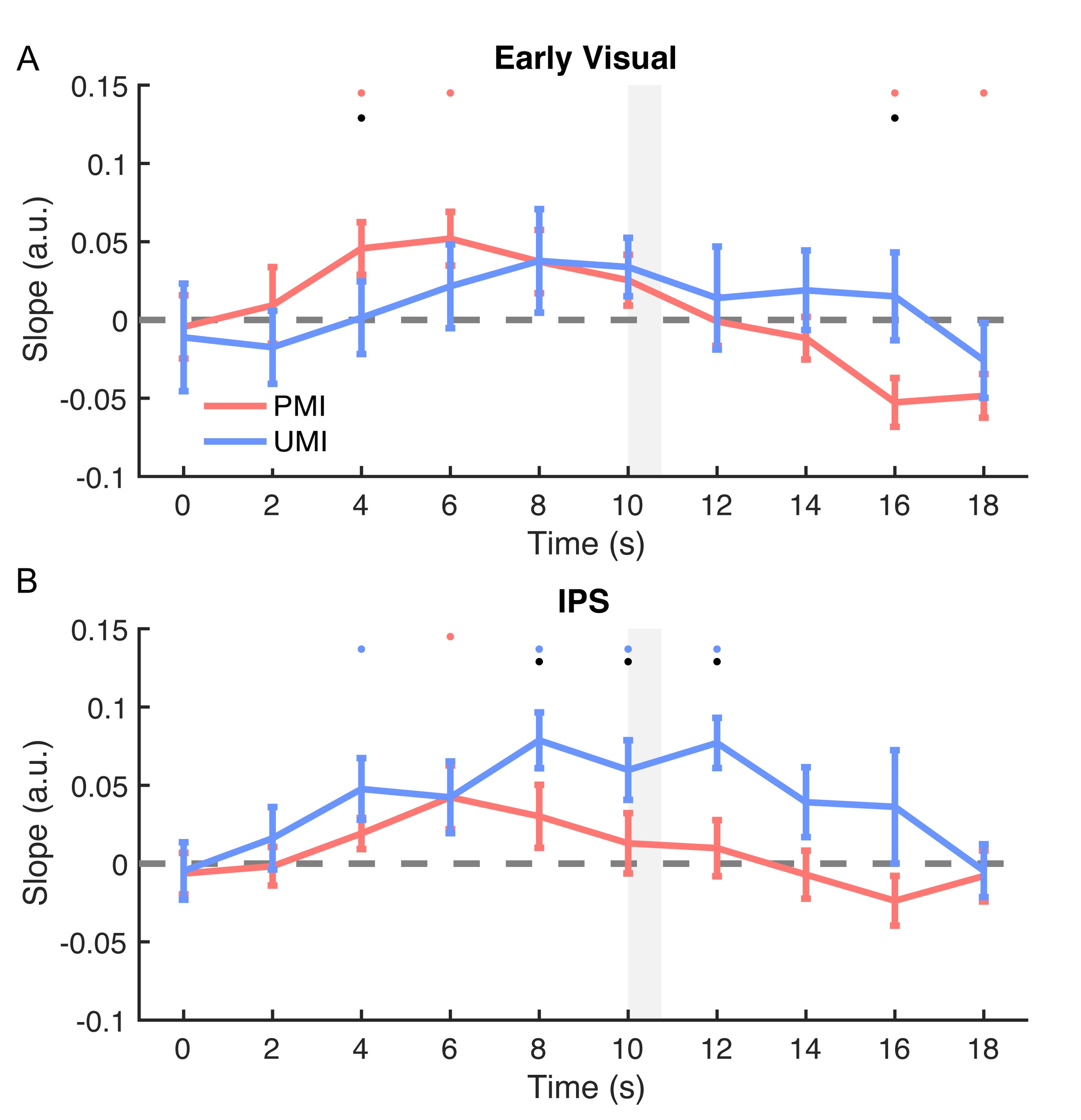

Supplement: S1 Fig — (A) Time course of the slope of orientation reconstructions in early visual ROI. (B) Time course of the slope of orientation reconstructions in IPS ROI. Slopes of the orientation reconstructions of the 2 sample items were plotted as a function of time from the beginning of the trial through the time point concurrent with the end of Delay1.2 and the onset of Recall1 (0–18 s after trial onset). All results were from UMI-trained IEMs. Red lines represent the PMI, and blue lines represent the UMI. Gray shaded area indicates display of Cue1 (10–10.75 s). Red, blue, and black dots indicate p < 0.05 for significant reconstruction of PMI, significant reconstruction of the UMI, and a significant difference between the two, respectively. All error bars indicate ± 1 SEM. Data are available at osf.io/G4C3N. IEM, inverted encoding model; IPS, intraparietal sulcus; PMI, prioritized memory item; ROI, region of interest; UMI, unprioritized memory item. (TIF) [file pbio.3000769.s001.tif]

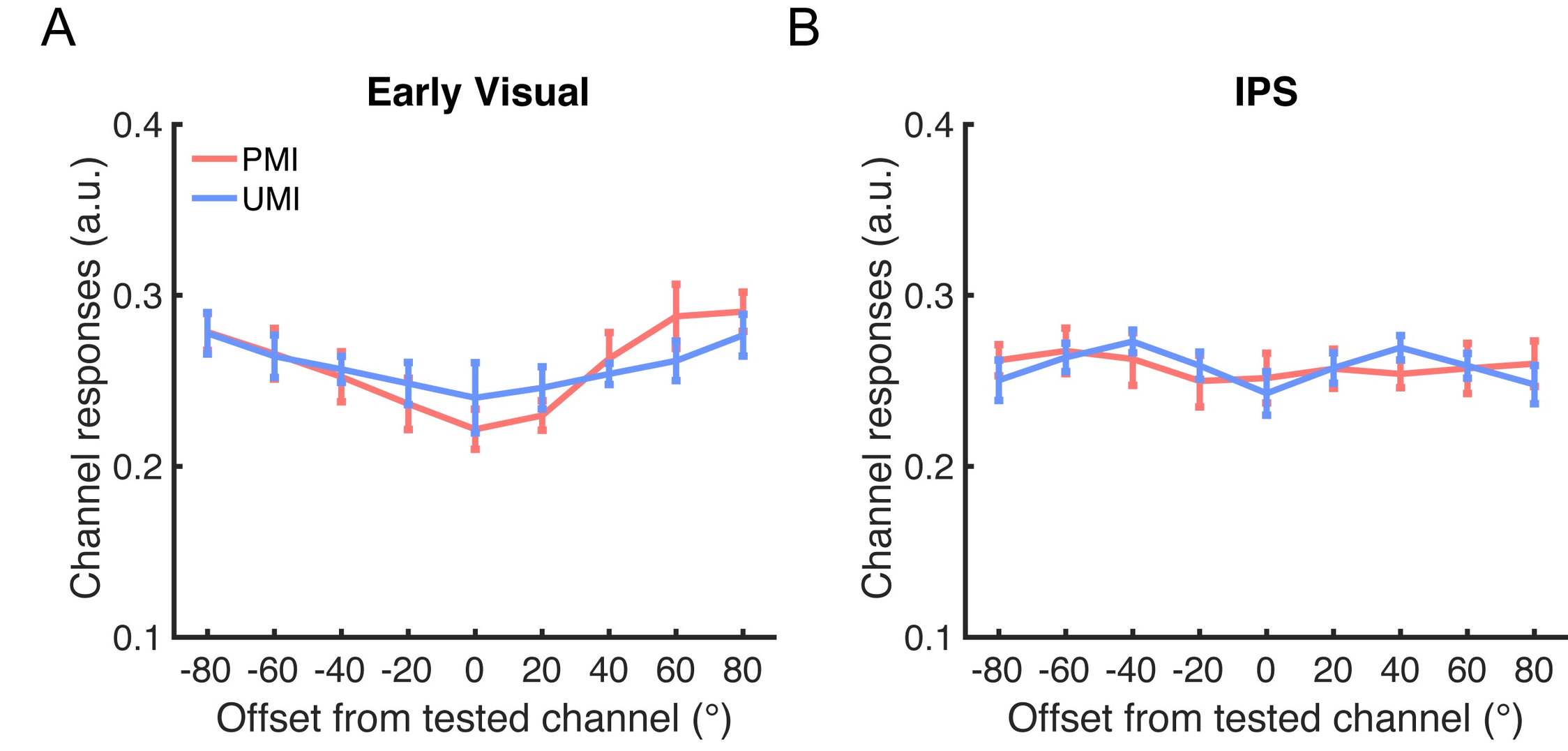

Supplement: S2 Fig — (A) IEM reconstructions of stimulus orientation during late Delay1.2 (18 s after trial onset) in early visual ROI. (B) IEM reconstructions of stimulus orientation during late Delay1.2 in IPS ROI. All results were from UMI-trained IEMs. Red lines represent the PMI, and blue lines represent the UMI. All error bars indicate ± 1 SEM. Data are available at osf.io/G4C3N. IEM, inverted encoding model; IPS, intraparietal sulcus; PMI, prioritized memory item; ROI, region of interest; UMI, unprioritized memory item. (TIF) [file pbio.3000769.s002.tif]

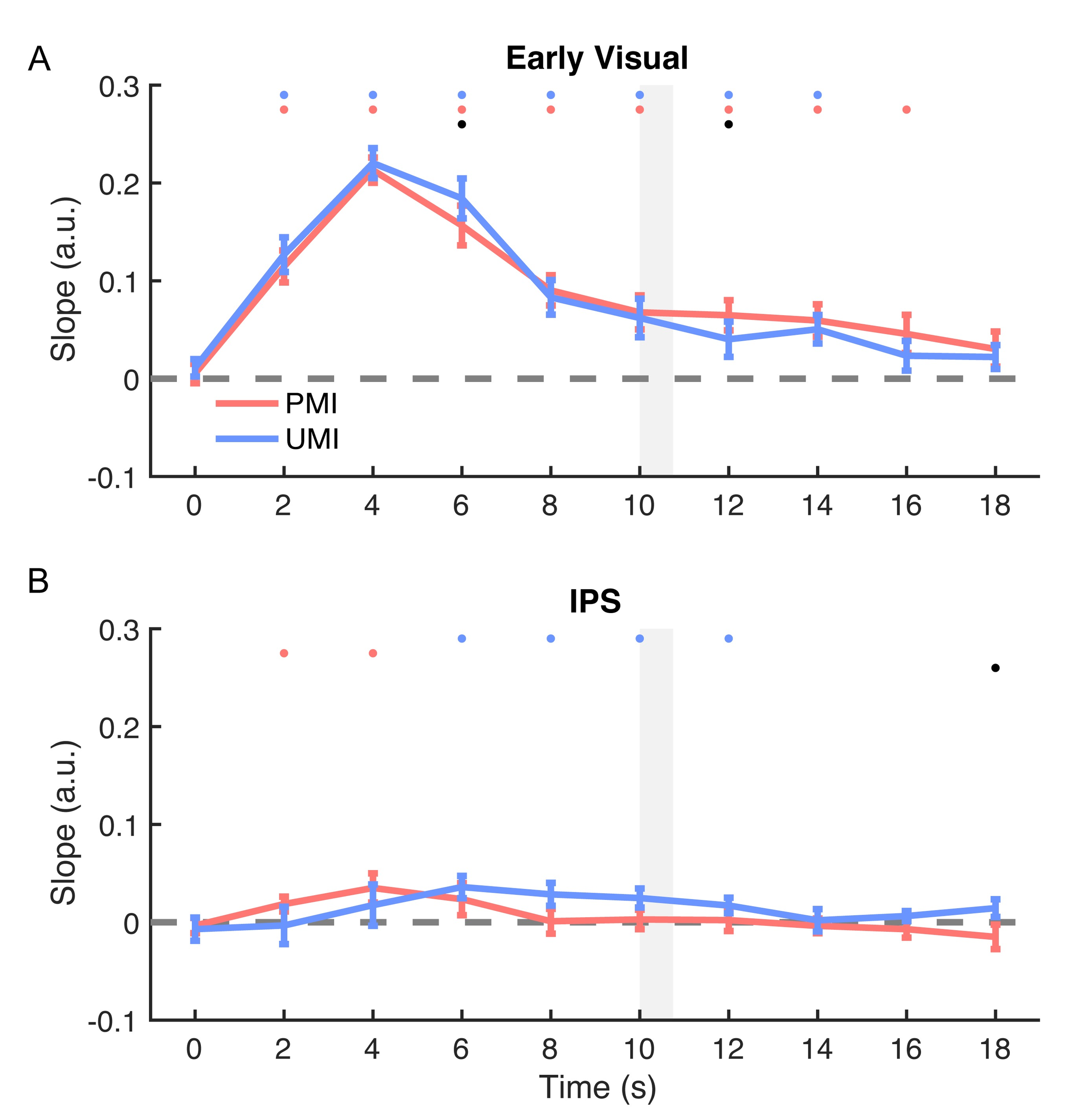

Supplement: S3 Fig — (A) Time course of the slope of location reconstructions in early visual ROI. (B) Time course of the slope of location reconstructions in IPS ROI. Slopes of the location reconstructions of the 2 sample items were plotted as a function of time from the beginning of the trial through the time point concurrent with the end of Delay1.2 and the onset of Recall1 (0–18 s after trial onset). All results were from UMI-trained IEMs. Red lines represent the PMI, and blue lines represent the UMI. Gray shaded area indicates display of Cue1 (10–10.75 s). Red, blue, and black dots indicate p < 0.05 for significant reconstruction of PMI, significant reconstruction of the UMI, and a significant difference between the two, respectively. All error bars indicate ± 1 SEM. Data are available at osf.io/G4C3N. IEM, inverted encoding model; IPS, intraparietal sulcus; PMI, prioritized memory item; ROI, region of interest; UMI, unprioritized memory item. (TIF) [file pbio.3000769.s003.tif]

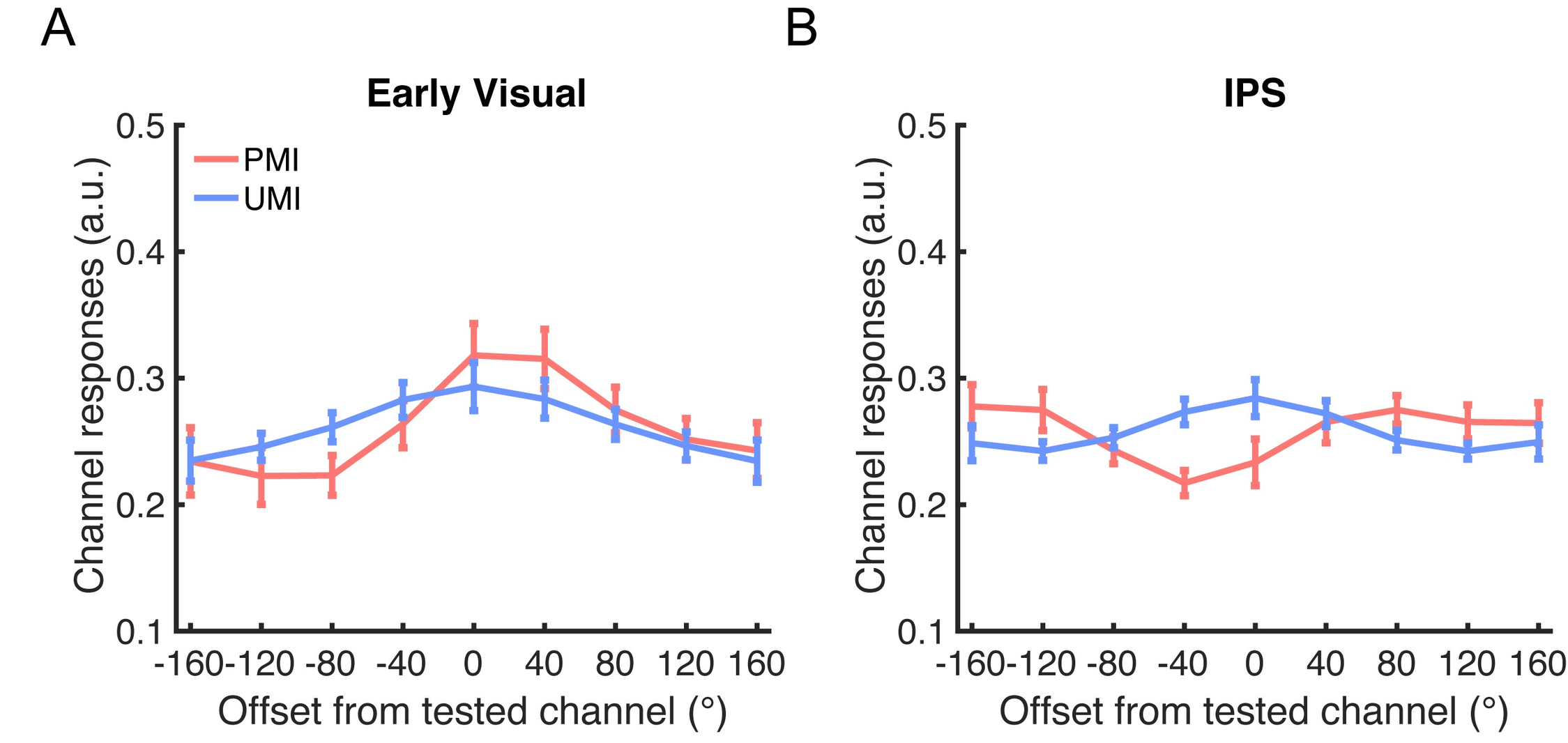

Supplement: S4 Fig — (A) IEM reconstructions of stimulus location during late Delay1.2 (18 s after trial onset) in early visual ROI. (B) IEM reconstructions of stimulus location during late Delay1.2 in IPS ROI. All results were from UMI-trained IEMs. Red lines represent the PMI, and blue lines represent the UMI. All error bars indicate ± 1 SEM. Data are available at osf.io/G4C3N. IEM, inverted encoding model; IPS, intraparietal sulcus; PMI, prioritized memory item; ROI, region of interest; UMI, unprioritized memory item. (TIF) [file pbio.3000769.s004.tif]

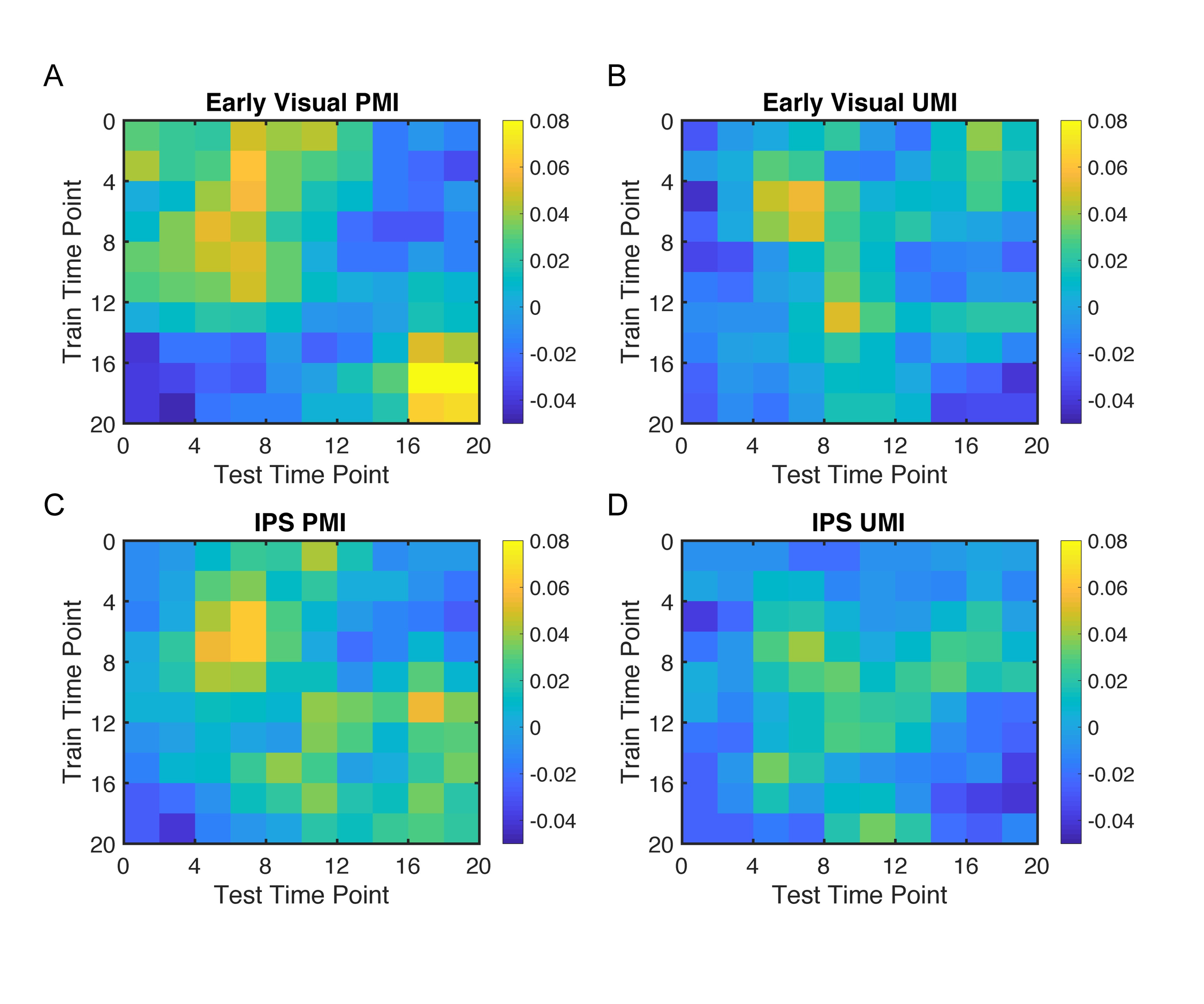

Supplement: S5 Fig — Temporal generalization of orientation reconstructions, in early visual and IPS ROIs, for PMIs and UMIs using PMI-trained IEMs. Strength of reconstructions are indicated by the slope of reconstructions. The x- and y-axes show the tested and training time points, respectively. Data are available at osf.io/G4C3N. IEM, inverted encoding model; IPS, intraparietal sulcus; PMI, prioritized memory item; ROI, region of interest; UMI, unprioritized memory item. (TIF) [file pbio.3000769.s005.tif]

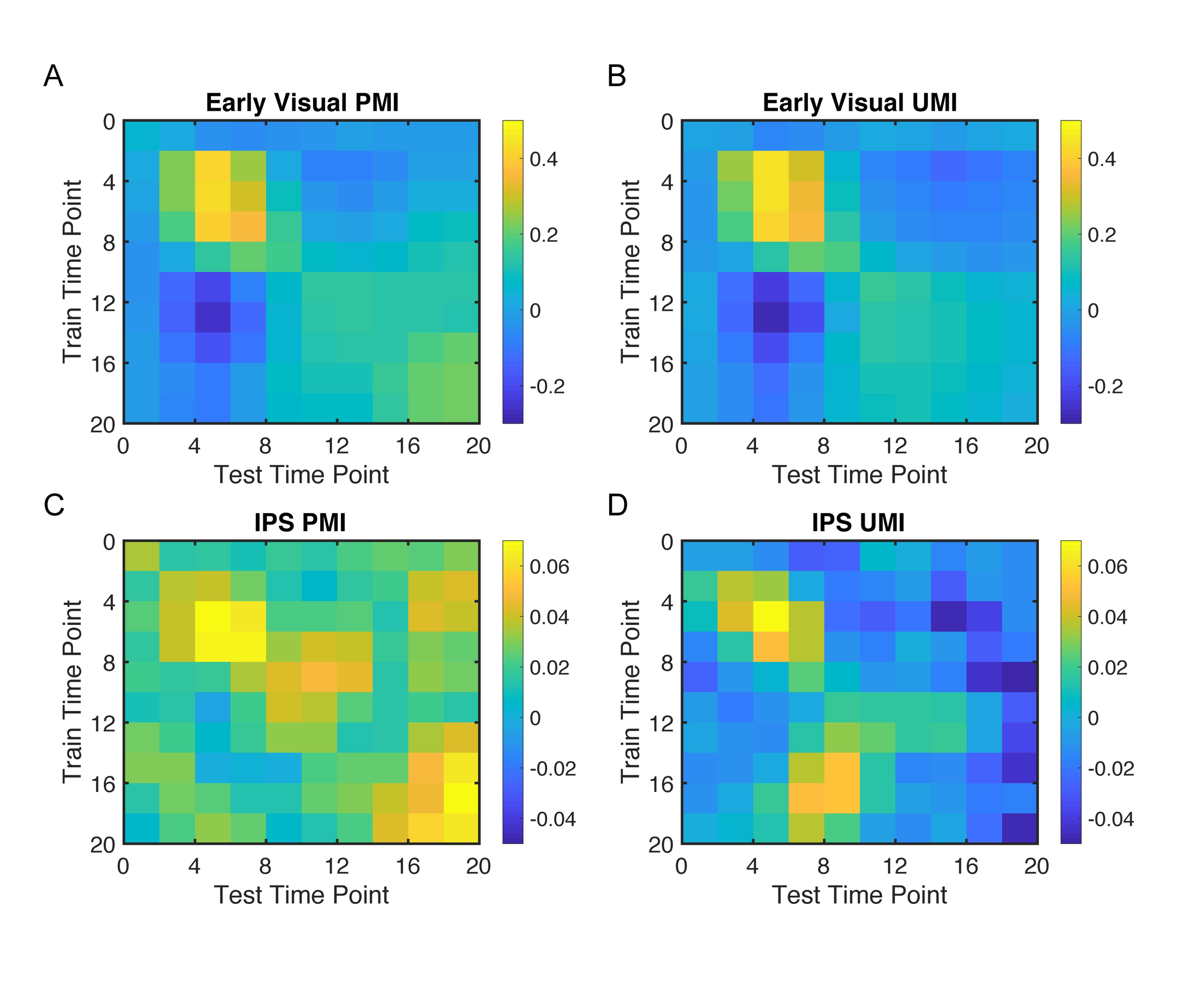

Supplement: S6 Fig — Temporal generalization of location reconstructions, in early visual and IPS ROIs, for PMIs and UMIs using PMI-trained IEMs. Strength of reconstructions are indicated by the slope of reconstructions. The x- and y-axes show the tested and training time points, respectively. Data are available at osf.io/G4C3N. IEM, inverted encoding model; IPS, intraparietal sulcus; PMI, prioritized memory item; ROI, region of interest; UMI, unprioritized memory item. (TIF) [file pbio.3000769.s006.tif]
